# Isl1 and Pou4f2 Form a Complex to Regulate Target Genes in Developing Retinal Ganglion Cells

**DOI:** 10.1371/journal.pone.0092105

**Published:** 2014-03-18

**Authors:** Renzhong Li, Fuguo Wu, Raili Ruonala, Darshan Sapkota, Zihua Hu, Xiuqian Mu

**Affiliations:** 1 Department of Ophthalmology/Ross Eye Institute, University of Buffalo, Buffalo, New York, United States of America; 2 Department of Biochemistry, University of Buffalo, Buffalo, New York, United States of America; 3 Department of Biostatistics, University of Buffalo, Buffalo, New York, United States of America; 4 Department of Medicine, University of Buffalo, Buffalo, New York, United States of America; 5 Center of Computational Research, New York State Center of Excellence in Bioinformatics and Life Sciences, University of Buffalo, Buffalo, New York, United States of America; 6 Developmental Genomics Group, New York State Center of Excellence in Bioinformatics and Life Sciences, University of Buffalo, Buffalo, New York, United States of America; 7 SUNY Eye Institute, University of Buffalo, Buffalo, New York, United States of America; 8 CCSG Cancer Genetics Program, Roswell Park Cancer Institute, Buffalo, New York, United States of America; National Eye Institute, United States of America

## Abstract

Precise regulation of gene expression during biological processes, including development, is often achieved by combinatorial action of multiple transcription factors. The mechanisms by which these factors collaborate are largely not known. We have shown previously that Isl1, a Lim-Homeodomain transcription factor, and Pou4f2, a class IV POU domain transcription factor, co-regulate a set of genes required for retinal ganglion cell (RGC) differentiation. Here we further explore how these two factors interact to precisely regulate gene expression during RGC development. By GST pulldown assays, co-immunoprecipitation, and electrophoretic mobility shift assays, we show that Isl1 and Pou4f2 form a complex in vitro and in vivo, and identify the domains within these two proteins that are responsible for this interaction. By luciferase assay, in situ hybridization, and RNA-seq, we further demonstrate that the two factors contribute quantitatively to gene expression in the developing RGCs. Although each factor alone can activate gene expression, both factors are required to achieve optimal expression levels. Finally, we discover that Isl1 and Pou4f2 can interact with other POU and Lim-Homeodomain factors respectively, indicating the interactions between these two classes of transcription factors are prevalent in development and other biological processes.

## Introduction

Retinal ganglion cells (RGCs) are projection neurons in the vertebrate retina whose axons form the optic nerve and project to the brain [1], [2]. During development, RGCs emerge from the mutipotent retinal progenitors cells [3]. In the mouse, RGC birth occurs between embryonic day (E) 11.5 to postnatal day (P) 0 and peaks at E14.5 [3], [4]. RGC development is subject to control by a hierarchical gene regulatory network in which key transcription factors occupy key nodes of the network [5], [6]. Three transcription factors, Math5, Isl1, and Pou4f2, in the network play essential roles in RGC development. Math5 is essential for RGC formation by rendering retinal progenitor cells competent for the RGC fate, and Isl1 and Pou4f2 (also known as Brn3b), although not required for RGC birth, are downstream of Math5 and required for their differentiation [5], [7], [8], [9], [10], [11], [12], [13].

Precise spatial and temporal gene expression is critical for normal development. This is mostly achieved via the combinatorial actions of multiple transcription factors. Interaction of Pou4f2 and Isl1 plays crucial roles in the precise expression of many genes in the developing RGCs. Pou4f2, a class IV POU domain transcription factor, and Isl1, a Lim-Homeodomain transcription factor, are co-expressed in developing RGCs [11], [12]. Knockout of either gene leads to severe, yet similar developmental defects of RGCs [11], [12]. RGCs in these knockout mice are born initially, but most of them die by apoptosis at later stages [9], [11], [12], [14]. Gene expression profiling analyses indicated that Isl1 and Pou4f2 regulate two distinct but overlapping sets of downstream genes in the developing RGCs [11]. Therefore, the similar defects in *Pou4f2*- and *Isl1*-null retinas seem to arise, at least in part, from the fact that they co-regulate a large set of downstream genes expressed in the RGCs. Nevertheless, how these two transcription factors collaborate to regulate these genes remains unknown.

Both the Lim-Homeodomain and POU domain families have large numbers of members [15], [16], [17] and interactions between members of these two families have been reported previously. In *C. elegans*, an analogous regulatory pathway to that defined by Pou4f2 and isl1 exists: MEC-3, a Lim-Homeodomain transcription factor, and UNC-86, a class IV POU domain transcription factor, are both required for the touch receptor neuronal fate. [18], [19], [20], [21]. They exert their roles by co-regulating downstream genes in a collaborative fashion by forming a complex on the DNA motifs of target genes [18], [19], [20], [21], [22], [23]. In *Drosophila*, Lim-Homeodomain proteins Tailup and Lim3 interact with the class III POU domain protein Drifter to specify the the ISNb motoneuron subclass [24]. In the mouse, Lim-Homeodomain protein Lhx3 and POU domain protein Pit1 function synergistically to regulate gene expression during pituitary gland development [25], [26]. In another case in the mouse, Isl1 and Pou4f1 (also known as Brn3a), another class IV POU domain transcription factor, collaborate to regulate gene expression in dorsal root ganglion neurons [27]. These reports indicate that collaboration between Lim-Homeodomain and POU domain transcription factors is common in diverse developmental processes. In most cases, however, the nature of interaction between these two families of transcription factors is not known.

In the current study, we explore how Isl1 and Pou4f2 collaborate to regulate gene expression in the developing RGCs. We find that these two transcription factors physically interact to form a complex, which can bind to DNA motifs of target genes. We further show that Isl1 and Pou4f2 contribute quantitatively to the optimal levels of expression of downstream genes. Moreover, we find both Isl1 and Pou4f2 interact with other POU and Lim-Homeodomain transcription factors respectively, indicating that physical interactions of these two families of transcription factors are evolutionarily conserved and play important roles in development.

## Materials and Methods

### Ethics statement

This study was carried out in strict accordance with the recommendations in the Guide for the Care and Use of Laboratory Animals of the National Institutes of Health. The protocol was approved by IACUCs of Roswell Park Cancer Institute and University at Buffalo (Protocol Number: 1147 M).

### Animals

The *Pou4f2*-null and floxed *Isl1* (*Isl1^flox^*) alleles have been described before [9], [11]. *Isl1*-null retinas were obtained by crossing *Isl1^flox^* with the *Six3-Cre* line [28]. These lines were kept in C57/BL6x129 mixed background. To collect embryos or embryonic retinas for in situ hybridization, total RNA isolation, and chromatin immuneprecipitation (ChIP), time-mated females at desired date of pregnancy were euthanized by CO2 inhalation, and the embryos or embryonic retinal tissues were harvested after anesthesia by cooling them on ice and decapitation.

### Construction of expression plasmids and protein expression and purification

Glutathione s-transferase (GST)-Pou4f2 (GST-P) fusion construct was made by cloning the Pou4f2 open reading frame (ORF) into the EcoR I and Xho I sites, in frame with the GST coding region, of pGEX-4T-1 (Life Technologies). GST-Isl1 fusion construct was made by cloning the Isl1 ORF into BamH I and Xho I sites, in frame with the GST coding region, of the pGEX-4T-3 vector (Life Technologies). Expression constructs for full-length Pou4f2 and its truncates, full-length Isl1 and it truncates, full-length Lhx2, full-length-Lhx1, and full-length Pou6f1 were made by cloning the coresponding coding region into the Nco I and Xho I sites of the pET-28a(+) vector (Life Technologies), in frame with the downstream His tag coding sequences, so that all these proteins were His-tagged. Expression construct for full-length Isl2 was made by cloning the Isl2 ORF into the Nco I and Not I sites of pET-28a(+). Expression constructs for full-length Pou4f3 and Pou3f2 (His-tagged) were made by cloning their ORFs into the BspH I and Not I sites of pET-28a(+) vector. Eukaryotic expression vectors expressing Isl1 or Pou4f2 were made by cloning their full-length cDNA into the EcoR I and Xho I sites of the pIRES-hrGFP-1a plasmid (Agilent). A construct expressing both Isl1 and Pou4f2 was made by cloning the ORFs of Isl1 and Pou4f2 together into the pIRES-hrGFP-1a plasmid, with the coding sequences of Isl1 and Pou4f2 separated by the coding sequences for the self-cleaving peptide T2A [29].

### GST pull-down assay

Recombinant protein expression plasmids were transformed into various *E. Coli* strains (BL21-CodonPlus (DE3)-RIPL, Rossetta 2 or Rosetta (DE3)pLysS). For protein expression, the transformed cells were induced with 0.4 mM IPTG for 2 hours at 37 °C and bacterial pellets were sonicated in the lysis buffer (50 mM Tris-HCl, pH 7.5, 100 mM NaCl, 1 mM DTT, 1 mM EDTA, 1 mM PMSF, 2 μg/ml leupeptin, 1 μg/ml pepstatin, 2 μg/mL aprotinin, 0.15% NP40, 10% glycerol). Supernatants were examined by SDS-PAGE and coomassie bright blue staining for evaluation of expression, and then used for GST pull-down assay. 500 μl of binding mixture containing about 20 μg of GST or GST-fusion lysates and 20 μg of the non-fusion protein lysates (125 mM Tris-HCl, 150 mM NaCl, pH 8.0) was incubated for 3∼4 hours at 4°C. 20 μl glutathione magnetic beads (Pierce, 66621) were then added into the mixture and incubated for at least 5 hours at 4°C, and washed 6 times with the binding buffer. The bound proteins were analyzed by 12% SDS-PAGE and Western blotting.

### Co-immunoprecipitation

HEK 293 cells were cultured at 37 °C with 5% CO_2_ in Dulbecco's modified Eagle's medium (DMEM) supplemented with 10% fetal calf serum (FCS, Atlanta Biologicals, Inc., GA) and antibiotics. Transfection was carried out with FuGENE HD (Promega, E2311) following the manufacturer's instructions. HEK 293 cells in a 10-cm plate were transfected with constructs expressing Isl1 and/or Pou4f2. Cell lysates were prepared 48 hours later with 600 μL of co-immunoprecipitation lysis buffer (phosphate-buffered saline, pH 7.4, containing 15% glycerol,10 mM DTT, and 10 mM NaF, 2 mM Na_3_VO_4_, proteinase inhibitor mixture, 1 mM PMSF) by sonication. The supernatants (1 mg of total proteins) were used to incubate with a rabbit polyclonal anti-Isl1 (Millipore) or rabbit IgG (Santa Cruz Biotechnology,) in 500 μl of co-immunoprecipitation lysis buffer for 3∼4 hours at 4°C. Protein A Dynabeads (Invitrogen, 10001D) (50 μl slurry for each sample) were washed 5 times with 1 ml of blocking buffer (PBS, 10% glycerol, 0.5% BSA), followed by incubation with the blocking buffer for 60 minutes at 4°C. The BSA-blocked Protein A Dynabeads were then added to the lysates/antibody mixture and further incubated at 4°C overnight. After 5 washes with co-immunoprecipitation lysis buffer, the proteins bound to beads were released by boiling in 70 μl 1× Laemmli protein sample buffer (Bio-Rad, 161-0747) for 10 minutes, and were analyzed by Western blotting with goat anti-Pou4f2 and mouse anti-Isl1.

### Antibodies

Antibodies used for Western blotting in this study include: monoclonal mouse anti-Isl1 (DSHB, 39.4D5, 1∶1000); polyclonal goat-anti-Isl1 (R&D systems, AF1837 1∶1000); polyclonal rabbit anti-Lhx1 (Santa-Cruz, sc-133735 1∶500); polyclonal goat anti-Lhx2 (Santa-Cruz, sc-19342, 1∶1000); polyclonal goat anti-Pou4f2 (Santa-Cruz, sc-6026, 1∶1000); polyclonal rabbit anti-GST (Sigma, G7781, 1∶1000); and monoclonal mouse anti-His (Santa-Cruz, sc-8036, 1∶500). Antibodies used for immunoprecipitation (IP) ChIP include polyclonal rabbit anti-Is1 (Millipore, NG1897037) and polyclonal goat anti-Pou4f2 (Santa-Cruz, sc-6026).

### Electrophoretic mobility shift assay (EMSA)

Isl1, Pou4f2, and their truncated versions were induced as described above. After sonication, the supernatants were loaded onto His SpinTrap columns (GE Healthcare, 28-4013-53) and after washing with washing buffer (20 mM sodium phosphate, 500 mM NaCl, 20 mM imidazole, pH 7.4), the His-tagged proteins were eluted with elution buffer (20 mM sodium phosphate, 500 mM NaCl, 500 mM imidazole, pH 7.4) following instructions of the manufacturer.

EMSA was performed as previously described [10], [30]. Double-stranded wild-type (W) and mutated oligonucleotides (M) were synthesized (see below) and radiolabeled by fill-in reaction with Klenow fragments. The binding reactions (20 mM Tris-HCl, pH 7.5, 0.05 M NaCl, 10 mM DTT, 1 mM EDTA and 5% glycerol) contained 20,000 cpm of labeled probe, 200 ng of dI/dC as non-specific competitor, and 200 ng of each recombinant protein in a total volume of 20 μl. The reactions were incubated at 4°C for 30 minutes. The protein-DNA complexes were resolved on an 8% non-denaturing polyacrylamide (acrylamide/bisacrylamide ratio of 37.5∶1) gel with 1xTBE (45 mM Tris borate, 1 mM EDTA, pH 8.0) as running buffer. Gels were pre-run for 1 hour at 145 V at 4°C and electrophoresis was allowed to proceed for 4 hours at 145 V at 4°C. The gels were then dried and autoradiographed.

The probe sequences included: SBRN3(W) Upper: 5′-GCA CAC GAC CCA ATG AAT TAA TAA CCG GGC TG-3′; SBRN3(W) Lower: 5′-GCA GCC CGG TTA TTA ATT CAT TGG GTC GTG TG-3′; SBRN3(M) Upper: 5′-GCA CAC GAC CCA GCG CGT TGA CAA CCG GGC TG-3′; SBRN3(M) Lower: 5′-GCA GCC CGG TTG TCA ACG CGC TGG GTC GTG TG-3′; CBRN3(W) Upper: 5′-GAT CTC TCC TGC ATA ATT AAT TAC CCC CGG AT-3′; CBRN3(W) Lower: 5′-GAT CCG GGG GTA ATT AAT TAT GCA GGA GAG AT-3′; CBRN3(M) Upper: 5′-GAT CTC TCC TGC GCG GTT GAC TAC CCC CGG AT-3′; CBRN3(M) Lower:5′-GAT CCG GGG GTA GTC AAC CGC GCA GGA GAG AT-3′;EBF3(W) Upper: 5′-GTG CGT TAA TAT TTT AAT TAA TCG GGA AAA-3′; EBF3(W) Lower: 5′- TTT TCC CGA TTA ATT AAA ATA TTA ACG CAC -3′; EBF3(M) Upper: 5′-GTG CGT TGC TAT TTT CGT TGC TCG GGA AAA-3′; EBF3(M) Lower: 5′-TTT TCC CGA GCA ACG AAA ATA GCA ACG CAC-3′; IRX6(W) Upper: 5′-GTA CTA CTA ATT AAT TAA TTG CAA ACA TTT C-3′; IRX6(W) Lower: 5′-GGA AAT GTT TGC AAT TAA TTA ATT AGT AGT A-3′; IRX6(M) Upper: 5′-TAC TAC TGC TTC GTT GCT TGC AAA CAT TTC; IRX6(M) Lower: 5′-GGA AAT GTT TGC AAG CAA CGA AGC AGT AGT A-3′.

### RNA-seq analysis

The whole data set will be published elsewhere. Total RNA was purified from E14.5 wild-type, *Pou4f2*-null, and *Isl1*-null retinas. E14.5 retinas were excised in ice-cold PBS, treated overnight at 4°C with RNAlater solution (Ambion, AM7020), and stored at −80°C. Eight to ten retinas of each genotype were pooled, and total RNA was isolated/purified using RNeasy Mini Kit (Qiagen, 74104) following the manufacturer's instructions. On-column digestion of DNA with RNase-Free DNase Set (Qiagen, 79254) was included in the purification protocol to ensure complete removal of DNA. Quality of the purified RNA was ensured by agarose gel electrophoresis, spectrophotometry, and BioAnalyzer analysis (Agilent, G2940CA). Three independent RNA samples were prepared for each genotype. The RNA samples were then used to generate Illumina sequencing libraries with TruSeq RNA Sample Prep Kit v2 kit (Illumina, RS-122-2001) following the manufacturer′s instruction and sequenced on an Illumina Hiseq 2000 sequencer. The sequence reads were then mapped to the mouse genome by Bowtie [31], and differentially expressed genes were then identified by Cufflinks 2 [32] following the authors′ instruction. Significance of differences in gene expression levels was also assessed by two-tailed student′s t test. All the sequences have been deposited into the NIH Short Read Archive (accession numbers SAMN02614558-SAMN02614569).

### Identification of potential Pou4f2 and Isl1 binding sites

Potential Pou4f2 and Isl1 binding sites were identified using the Whole Genome rVISTA web portal [33], [34], which identifies transcription factor binding sites that are conserved between species and over-represented in upstream regions of groups of genes. The input genes were those that were down-regulated in either *Pou4f2*- or *Isl1*-null retinas, and 5000 bp upstream sequences were analyzed. These analyses identified enrichment of binding sites for Pou4f2 and Isl1 within these genes, which were further inspected manually to identify overlapping sites that could be potentially recognized by both Isl1 and Pou4f2 for experimental analysis.

### ChIP

ChIP with embryonic retinal tissues was performed following a previously published protocol with minor modifications [35]. Approximately 100 E14.5 mouse retinas were collected, cross-linked with 1% paraformaldehyde in PBS for 18 minutes at room temperature. The tissues were then lysed with lysis buffer (20 mM Tris-HCl, pH 8.0, 85 mM KCl, 0.5% NP-40, Proteinase Inhibitors) and the nuclei were harvested and lysed with nuclear lysis buffer (50 mM Tris-HCl pH 8.0, 10 mM EDTA, 1% SDS, proteinase Inhibitors). The chromatins were then sonicated to break the DNA to ∼200 to 1,000 bp in length, and then incubated with the desired antibody (goat anti-Isl1, goat anti-Pou4f2 or goat IgG) overnight at 4°C. The chromatin/antibody mixture was then incubated with magnetic Protein A beads for 4 hours at 4°C. The beads were then collected and thoroughly washed with RIPA buffer (10 mM Tris-HCl, pH 8.0,1 mM EDTA, 0.5 mM EGTA, 140 mM NaCl, 1% Triton X-100, 0.1% Na-deoxycholate, 0.1% SDS). The precipitated DNA chromatins were then eluted, digested with proteinase K, and the DNA was then purified with QIAquick PCR purification kit (Qiagen, 28104). The DNA was eluted in 30 μl water. One to two μl of the DNA was used for PCR with primers flanking the region of interest. PCR reactions were performed as follows: pre-denaturing at 95°C for 10 minutes, followed by 40 cycles of denaturing at 94°C for 30 seconds, annealing at 55°C for 30 seconds, and extension at 72°C for 40 seconds, and a final chase at 72°C 15 minutes. The primers used included: Ebf3 Forward: 5′-GGT GGT GTG TGT GCG TTA ATA TTT T-3′; Ebf3 Reverse: 5′-GCT TTG ATA ATT CAC TTA TCT TTG G-3′;Irx6 Forward: 5′-GAT GTT GCT AAG CTC TCT CCC TCT T-3′;Irx6 Reverse: 5′-ATT TGC TGA GTA AGT ATT TCA ACT A-3′; β-actin Forward: 5′-ATC ATG TTT GAG ACC TTC AAC A-3′; β-actin Reverse: 5′-GGT CAG GAT CTT CAT GAG GTA-3′.

### In situ hybridization

In situ hybridization was performed as previously described [10], [11]. Briefly, E14.5 embryos of different genotypes were paraffin embedded, sectioned at 10 μM, and de-waxed. Anti-sense probes were labeled by T3 RNA polymerase with DIG by in vitro transcription. Hybridization incubations were carried out in hybridization buffer [50% formamide, 5×SSC (pH 4.5–5.0), 1% SDS, 50 μg/ml yeast tRNA, 50 μg/ml heparin] at 65°C overnight, washed three times with wash buffer [50% formamide, 1×SSC (pH 4.5–5.0), 1% SDS] at 65°C. The slides were then incubated with alkaline phosphatase-conjugated anti-DIG antibody (Roche, 11093274910) in 1xMABT (100 mM maleic acid, 150 mM NaCl, 0.1% Tween 20, pH 7.5) overnight at room temperature. The slides were developed by incubation with BM Purple (Roche, 11442074001) at room temperature until the signals reach desired intensities.

### Cell culture, transfection and luciferase assay

HEK 293 cells were cultured in DMEM with 10% FBS at 37°C with 5% CO_2_. The luciferase reporter construct containing three consensus Pou4f2 binding sites and the minimal rat prolatcin promoter (-36PRL) was reported previously [10], [36]. This construct was cotransfected with Pou4f2 and/or Isl1 expressing constructs.Transfection was carried out in six-well plates using FuGENE HD (Promega, E2311) following the manufacturer's protocol. For each transfection, 10 ng of reporter plasmid, 1 μg of Pou4f2 and/or Isl1 expression plasmid or empty vector, and 1 ng of pRL-CMV (Promega) expressing Renilla luciferase were used. Three transfection replicates were performed for each individual experiment. Cells were harvested 36 hours after transfection, and luciferase activities were measured by the Dual-Luciferase Reporter Assay System (Promega, E1910). To control for variations in transfection efficiency among wells, the firefly luciferase activities were normalized to Renilla luciferase activities. Significance of differences was assessed by two-tailed student's t test assuming equal variance.

## Results

### Isl1 and Pou4f2 interact physically

**Figure 1:**
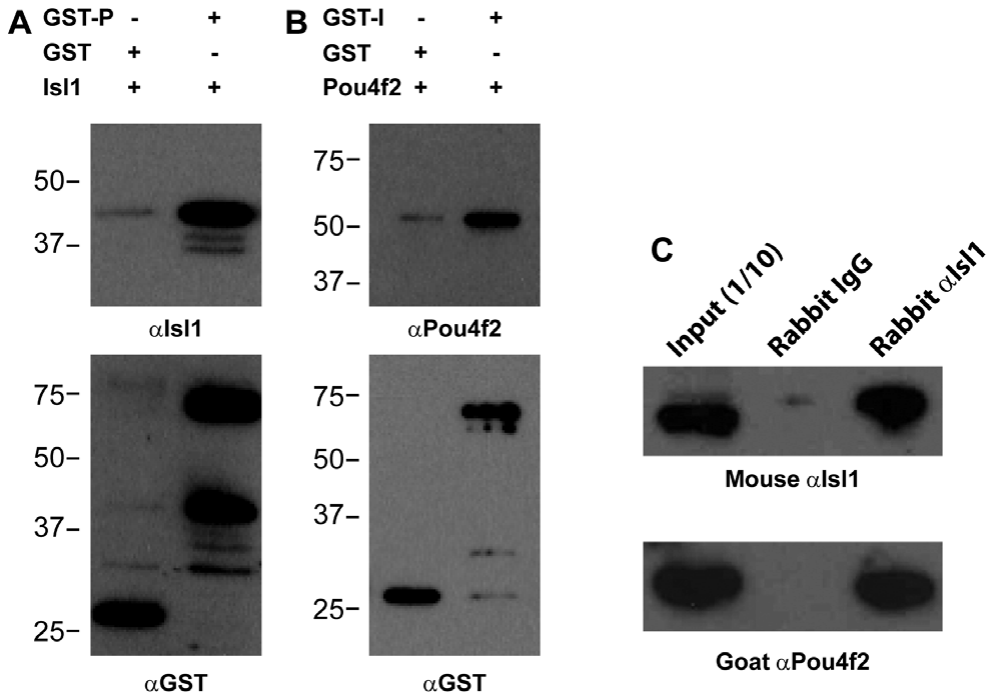
Pou4f2 and Isl1 form a complex in vitro and in vivo. A. Top: GST-Pou4f2 fusion protein (GST-P), but not GST, can interact with Isl1, as detected by anti-Isl1. Bottom: anti-GST demonstrates the GST and GST-Pou4f2 proteins used in the pull-down assays. Smaller bands in the GST-Pou4f2 lane are degradation products. B. Top: GST-Isl1 fusion protein (GST-I), but not GST alone, can interact with Pou4f2 as detected by anti-Pou4f2. Bottom: anti-GST recognizes both GST and GST-Isl1. Like GST-Pou4f2, there was also degradation of GST-Isl1 as indicated by the smaller bands. C. Co-Immunoprecipitation (IP) of Isl1 and Pou4f2 by a rabbit anti-Isl1. Both proteins were expressed in the HEK293 cells and could be detected in the input lysate by a mouse anti-Isl1 and goat anti-Pou4f2. In the rabbit IgG control, neither Isl1 nor Pou4f2 could be detected. When rabbit anti-Isl1 was used for the IP, both Isl1 and Pou4f2 could be detected.

To explore the mechanism of Isl1/Pou4f2 collaboration, we first used GST pull-down assay to investigate whether these two proteins can directly interact with each other. For this purpose, we expressed the two proteins either alone or in fusion with GST in *E. Coli*. Mutual GST pull-down assays were then performed with *E. coli* lysates containing GST-Pou4f2 and Isl1, or GST-Isl1 and Pou4f2. As shown in **Fig. 1A**, Isl1, as detected by anti-Isl1, was pulled down by GST-Pou4f2, but not by GST alone. Conversely, GST-Isl1, but not GST, pulled down Pou4f2 (**Fig. 1B**). These results indicated that Isl1 and Pou4f2 can physically interact and form a complex in vitro.

Next we examined whether Isl1 and Pou4f2 could form a complex within the cell by co-immunoprecipitation (co-IP). Since embryonic retinas are very small, it was not practical to obtain enough embryonic retinal tissues to perform the experiment. To get around this, we co-expressed Pou4f2 and Isl1 in HEK-293 cells and used a rabbit anti-Isl1 to precipitate Isl1 from the cell lysates. The immunoprecipitated proteins were then detected by Western blotting for the presence of Isl1 and Pou4f2. The rabbit anti-Isl1 antibody, but not IgG, efficiently precipitated Isl1, as detected by a mouse anti-Isl1 antibody on Western blot (**Fig. 1C**). When the same precipitated elutants were detected by anti-Pou4f2, a strong band was detected in elutants from anti-Isl1, but not from control IgG. These results suggest that, in agreement with the in vitro GST pull-down assays, Isl1 and Pou4f2 do physically interact to form a complex in vivo.

These results are consistent with our previous findings that the two transcription factors co-regulate a substantial set of genes in the developing RGCs and suggest the mechanism by which Isl1 and Pou4f2 co-regulate these genes is through physical interaction.

### Distinct domains of Isl1 and Pou2 are involved in their interaction

**Figure 2:**
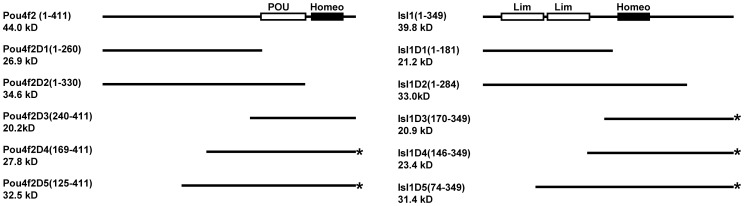
Diagram of serial deletions of Pou4f2 and Isl1. Diagram of the Pou4f2 (Left) and Isl1 (right) protein structures and the truncated deletions we expressed in *E. Coli*. The amino acid residues for each deletion and their estimated molecular weights are indicated. All proteins are His-tagged at the C terminus for easy detection by Western Blotting, although the Isl1 protein and its truncates are detected by a polyclonal rabbit anti-Isl1. * indicates constructs capable of protein-protein interaction (see **Figs. 3** **5**).

We then investigated what domains within the two proteins were responsible for the interaction of the two proteins. The mouse Pou4f2 protein has 411 amino acid (a.a.) residues, and the mouse Isl1 protein has 349 a.a. residues. The schematic structures of the two proteins are shown in **Fig. 2**. The Pou4f2 protein contains a POU-specific domain and a homeodomain in the C terminal region, whereas the Isl1 protein contains two Lim domains in the N terminal region and a homeodomain in the central region. We first made a series of truncated constructs for these two proteins and expressed them in *E. Coli* (**Fig. 2**). Although His-tags were added to all the constructs for easy detection by anti-His on Western blotting, only Pou4f2 truncated were detected by anti-His, and all the Isl1 constructs were detected by a rabbit polyclonal anti-Isl1 raised against the whole protein, since anti-His could not detect the Isl1 truncates efficiently for unknown reasons. For Pou4f2, the truncated versions included one missing both the POU and homeo domains (Pou4f2D1, a.a. 1–260), one missing just the homeodomain (Pou4f2D2, a.a. 1-330), and three missing varying lengths from the N terminus but maintaining the POU an homeo domains intact (Pou4f2D3, a.a. 240–411; Pou4f2D4, a.a. 169–411; Pou4f2D5, a.a. 125–411) (**Fig. 2** **, left**). For Isl1, the truncated versions included one missing the homeodomain and the C terminal region (Isl1D1, a.a. 1–181), one missing just part of the C terminal region (Isl1D2, a.a. 1–284), and three missing various lengths from the N terminus (disrupting or deleting one or both Lim domains) (Isl1D3, a.a. 170–349; Isl1D4, a.a. 146–349; Isl1D5, a.a. 74–349) (**Fig. 2** **, right**).

**Figure 3:**
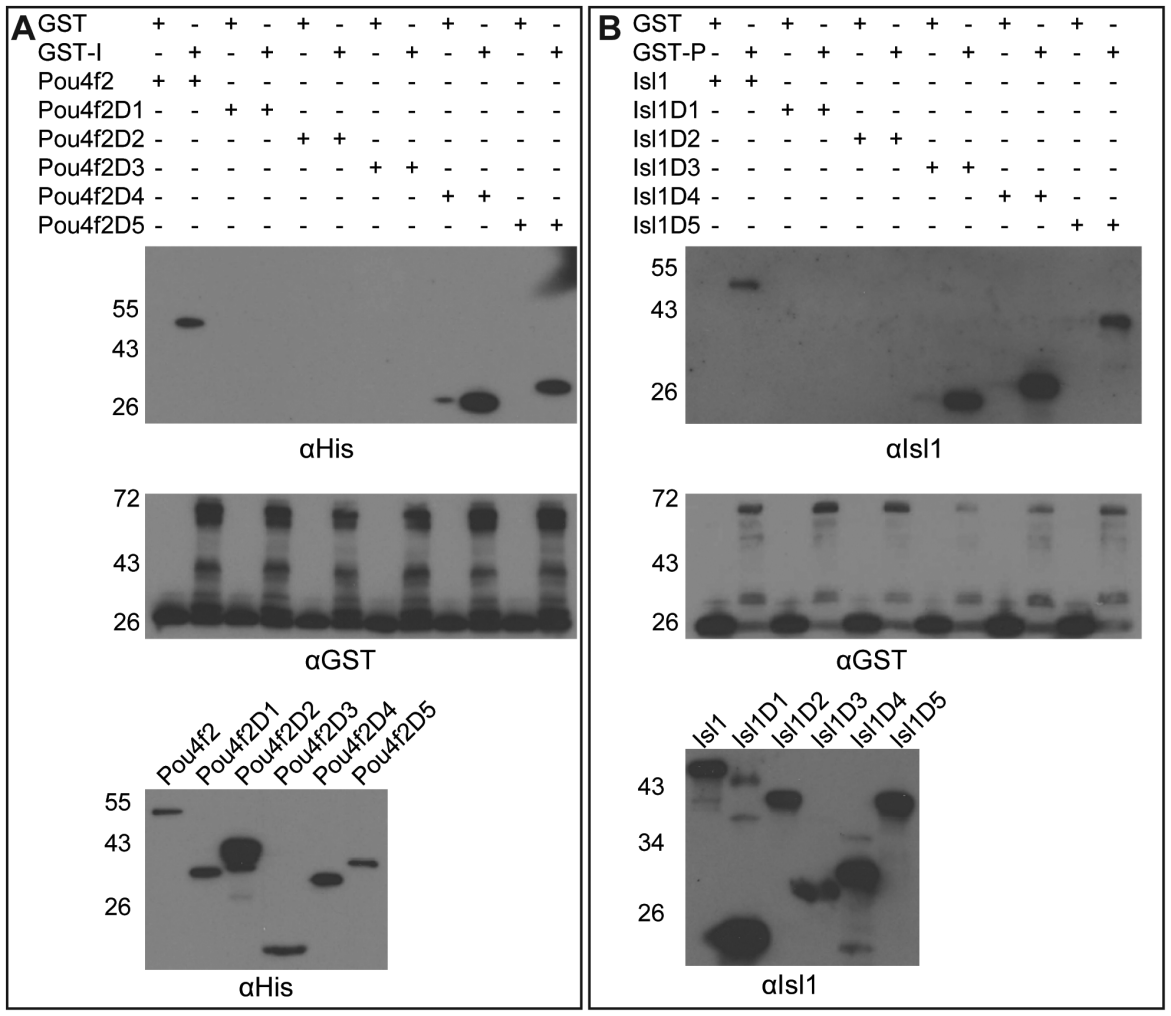
Identification of domains within Pou4f2 and Isl1 responsible for their mutual interaction. A. Top: GST-Isl1 could interact with Pou4f2D4 and Pou4f2D5, but not Pou4f2D1, Pou4f2D2 or Pou4f2D3, as detected by anti-His. Full-length Pou4f2 serves as positive control. Middle: Input GST or GST-Isl1 detected by anti-GST. Bottom: Input Pou4f2 and its deletions could all be detected by anti-His. B: Top: GST-Pou4f2 (GST-P), but not GST, could interact with Isl1, Isl1D3, Isl1D4 and Isl1D5, but not with Isl1D1 and Isl1D2. Middle: Input GST or GST-Pou4f2 as detected by anti-GST. Bottom: Input Isl1 or its deletions as detected by a rabbit polyclonal anti-Isl1. Sizes (Kda) of protein markers are indicated on the side. Antibodies used for each blot are indicated at the bottom of each panel.

We then performed GST pull-down assay with GST-Isl1 and the truncated Pou4f2 proteins, or with GST-Pou4f2 and truncated Isl1 proteins (**Fig. 3**). Other than the full-length Pou4f2, GST-Isl1 could also pull down Pou4f2D4 and Pou4f2D5, but not Pou4f2 D1, Pou4f2D2, or Pou4f2D3, indicating that, among the deletion proteins, Pou4f2D4 contains the minimal region responsible for the Isl1/Pou4f2 interaction (**Fig. 3A**). This suggested that the DNA binding domain, including the POU domain and the homeodomain, of Pou4f2 was involved in the interaction with Isl1 (**Fig. 2**). Consistent with this, Pou4f2D2 and Pou4f2D1, which lacked either the homeodomain or both the POU and homeo domains (**Fig. 2**), were incapable of interacting with Isl1. Nevertheless, Pou4f2D3, which contains both the POU and homeo domains (**Fig. 2**), could not interact with Isl1, indicating that the DNA-binding domain alone was not sufficient. Since the only difference between Pou4f2D3 and Pou4f2D4 was the 24 a.a. residues on the N terminal side of the DNA-binding domain (**Fig. 2**), these a.a. residues were also essential for the Isl1/Pou4f2 interaction. Thus, the region containing the DNA-binding domain along with a small stretch of a.a. residues on its N terminal side in the Pou4f2 protein is required for the interaction with Isl1.

When we used GST-Pou4f2 as bait, three truncated Isl1 proteins, Isl1D3, Isl1D4, and Isl1D5, were pulled down, but the other two truncates, Isl1D1 and Isl1D2 were not, indicating Isl1D3, Isl1D4, and Isl1D5, but not Isl1D1 or Isl1D2, interact with Pou4f2 (**Fig. 3B**). Among the three truncates interacting with Pou4f2, Isl1D3 (a.a.170-349) was the smallest (**Fig. 2**), containing only the homeodomain and the C terminal region, but none of the Lim domains. Our findings that both Isl1D1, which had both the homeodomain and the C terminal region deleted, and Isl1D2, which missed just part the C terminal region but not the homeodomain (**Figs. 2** **,** **3**), failed to be pulled down by GST-Pou4f2, indicated both regions were required for Isl1 to interact with Pou4f2. The Lim domains, which are involved in protein-protein interactions of Lim-Homeodomain proteins with many other partners [37], appeared not to be involved in the interaction of Isl1 with Pou4f2.

These results thus indicate the DNA-binding domains of both proteins are involved in the interaction with each other. However, the DNA-binding domains alone seem not to be sufficient. The C terminal region of Isl1 and the region adjacent to the POU domain of Pou4f2 on the N terminal side are also required for this interaction.

### Isl1 and Pou4f2 co-bind to specific DNA elements

**Figure 4:**
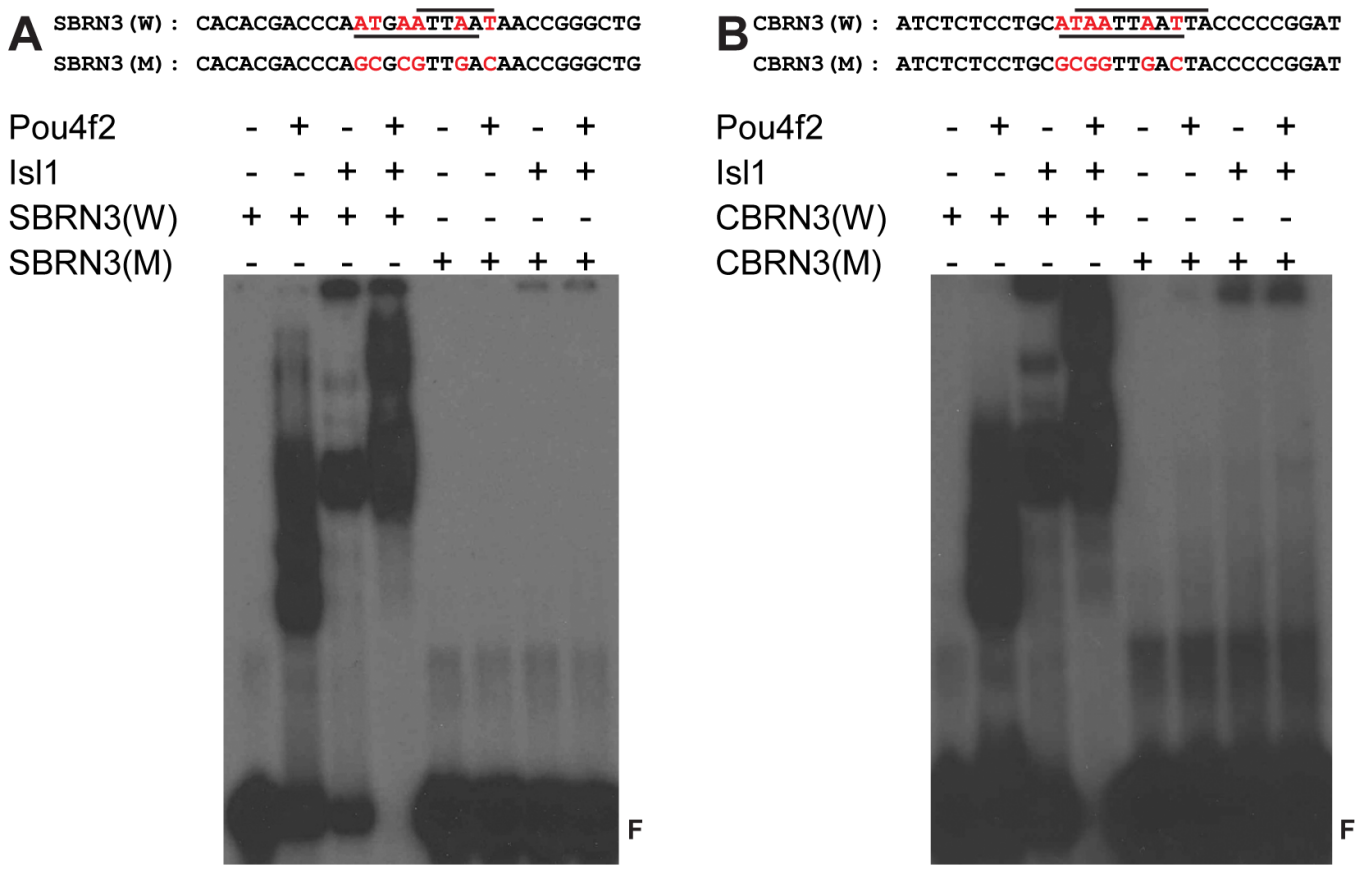
Pou4f2 and Isl1 bind to DNA elements individually and as a complex in EMSA. A: Wild-type (SBRN3(W)) sequence from the conserved element in the Sonic Hedgehog gene and its mutant sequence SBRN3(M) are shown on the top. Lines on top and beneath the SBRN3(W) sequence indicate the ATTA cores and Pou4f2 binding motif respectively. At Bottom, EMSA shows that either Pou4f2 or Isl1 alone can bind to SBRN3(W) as indicated by the slow migrating DNA-protein complexes, but not to SBRN3(M). When both Pou4f2 and Isl1 are present, they form a further slow-migrating DNA-protein complex with SBRN3(W), but not with SBRN3(M). B: Similarly, Pou4f2 or Isl1 alone binds to CBRN3(W), but not to CBRN3(M); Pou4f2 and Isl1 form a complex on CBRN3(W), but not on CBRN3(M). F indicates free probes. Lines on top and beneath the CBRN3(W) sequence indicate the ATTA cores and Pou4f2 binding motif respectively.

Since the DNA-binding domains of both Isl1 and Pou4f2 were involved in their interaction, we next investigated their ability to interact with DNA by EMSA using purified Isl1 and Pou4f2 proteins (**Fig. 4**). We used two DNA motifs recognized by Pou4f2, one found in a conserved region (SBRN3) in the first intron of the sonic hedgehog gene (*Shh*) [10] (**Fig. 4A**), and the other identified as consensus motif by in vitro SELEX (CBRN3) (**Fig. 4B**) [38]. As previously reported [10], Pou4f2 could bind with probes containing either motif efficiently (W), but not with those in which core nucleotides were mutated (M) (**Fig. 4**). Intriguingly, we noticed that the two motifs contained either two (SBRN3) or three (CBRN3) ATTA sequences, which are the core sequences of DNA motifs recognized by Isl1 [39], [40]. Isl1 can bind to DNA sequences with a single ATTA motif, but multiple motifs are required for high affinity binding [39], [40], although the spacing requirement between these motifs has not been thoroughly investigated. Consistent with this, Isl1 also could efficiently bind both probes alone, but again not with those containing the mutant sequences (**Fig. 4**). These observations suggested that at least some motifs recognized by Pou4f2 could also be recognized by Isl1. When both Pou4f2 and Isl1 were present, an even slower migrating complex was detected by EMSA with both the SBRN3 and the CBRN3 probes, but not with either mutant ones (**Fig. 4**). This indicated the Isl1/Pou4f2 complex still formed and bound to the DNA motif to form a ternary complex.

**Figure 5:**
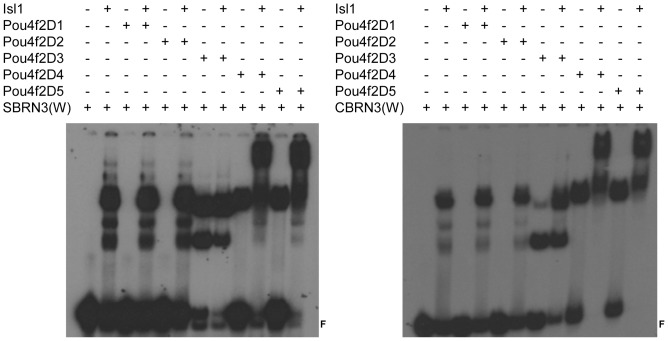
EMSA with Pou4f2 truncations and Isl1. Left: Isl1 forms complexes with Pou4f2D4 and Pou4f2D5 on SBRN3(W), but not with Pou4f2D1, Pou4f2D2 and Pou4f2D3. Right: Isl1 forms complexes with Pou4f2 D4 and Pou4f2D5, but not with Pou4f2D1, Pou4f2D2 and Pou4f2D3 on CBRN(W). F is free probe. These results are consistent with those from the pull-down assays.

Next we examined how the various deletions of Pou4f2 and Isl1 behaved in the presence of DNA by EMSA, using both the SBRN3 and CBRN3 probes (**Fig. 5** **,** **6**). Pou4f2D1and Pou4f2D2, which had part or the entire DNA binding domain deleted (**Fig. 2**), did not form complexes with the DNA probes as expected (**Fig. 5**). Further, their presence did not change the migration of complexes formed between full-length Isl1 and the two probes, indicating Pou4f2D1 and Pou4f2D2 did not form complexes with Isl1 in the presence of DNA. Pou4f2D3 had the DNA-binding domain intact and formed protein-DNA complexes with both probes (**Fig. 5**). However, when both Pou4f2D3 and Isl1 were present, no slower migrating complex was observed in EMSA, but the shifted bands resembled more like those by Pou4f2D3 alone, rather than those by Isl1 alone. This result could be interpreted as that Pou4f2D3 had higher affinities for the probes than Isl1, thus excluding Isl1 from binding to the probes, but it did not form a complex with Isl1 even in the presence of the DNA motifs. Pou4f2D4 and Pou4f2D5 could both form DNA complexes with the DNA probes by themselves (**Fig. 5**). When Isl1 was present, further slower migrating complexes were observed, indicating that Pou4f2D4 and Pou4f2D5 formed complexes with Isl1 on the DNA probes.

**Figure 6:**
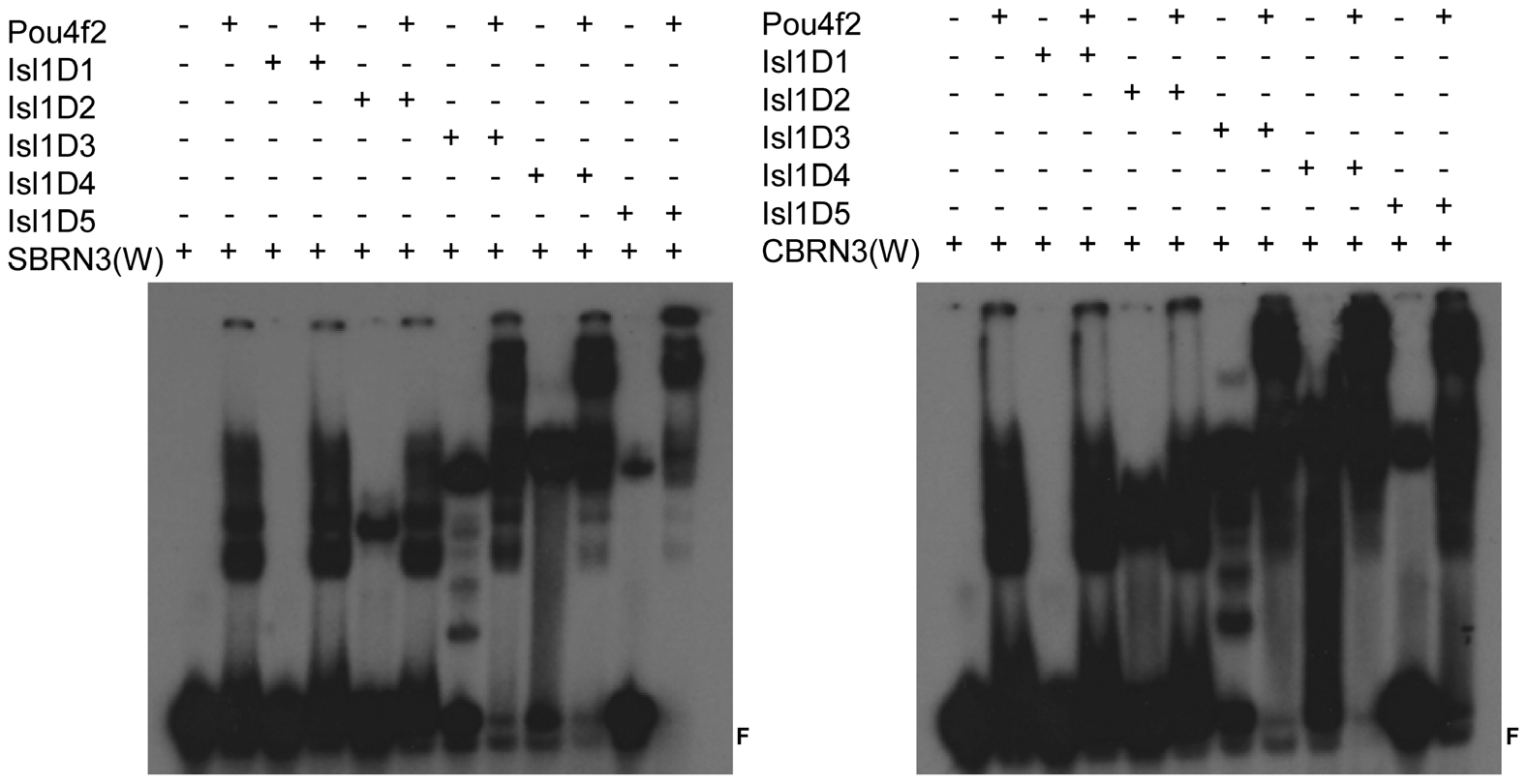
EMSA with Isl1 truncations and Pou4f2. Left: Pou4f2 forms complexes with Isl1D3, Isl1D4 and Isl1D5 on SBRN3(W), but not with Isl1D1 and Isl1D2. Right: Pou4f2 forms complexes with Isl1D3, Isl1D4 and Isl1D5 on CBRN3(W), but not with Isl1D1 and Isl1D2. F indicates free probes.

For Isl1, Isl1D1 did not have the DNA-binding homeodomain and, as expected, did not form a protein-DNA complex with the probes (**Fig. 6**). Its presence also did not affect the binding of Pou4f2 to the DNA probes. Isl1D2, which had the homeodomain intact, did form a DNA-protein complex. In the presence of both Isl1D2 and full-length Pou4f2, however, the DNA-protein complex was similar to that formed when Pou4f2 was present alone (**Fig. 6**), further supporting the idea that Pou4f2 has higher affinities than Isl1 for the DNA probes. Isl1D3, Isl1D4, and Isl1D5 all had the homeodomain intact and all could form protein-DNA complexes themselves with the SBRN3 and CBRN3 probes (**Fig. 6**). More significantly, when Pou4f2 was present, slower migrating complexes were observed for all three of them, indicating Isl1D3, Isl1D4, and Isl1D5 all formed complexes with Pou4f2 in the presence of the DNA motifs.

Thus, the same truncations of Isl1 or Pou4f2, which could form complexes with the other protein, formed ternary complexes with DNA. Based on these findings, we conclude that Isl1 and Pou4f2 form complex both in the presence and absence of specific DNA motifs, and the regions responsible for their interaction include the DNA-binding domains and adjacent sequences from both proteins.

### Isl1 and Pou4f2 co-regulate target genes by binding to the same cis elements

**Figure 7:**
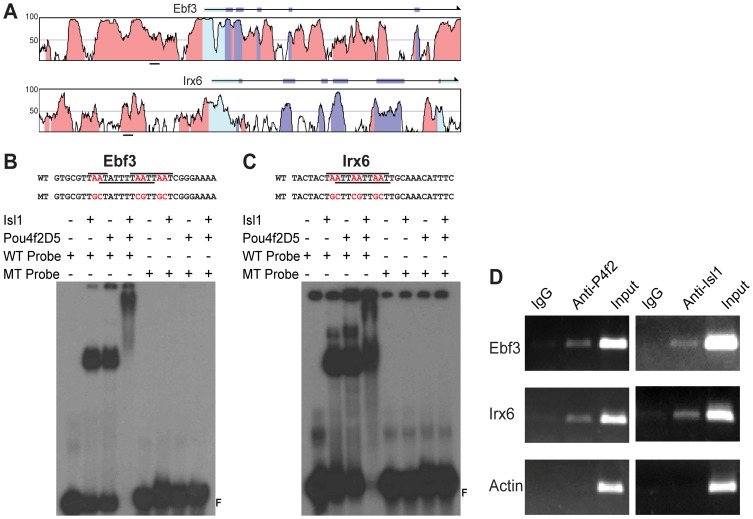
Conserved DNA motifs in *Ebf3* and *Irx6* are recognized by Isl1/Pou4f2 complex. A: Mouse-human comparison to identify conserved regions in *Ebf3* and *Irx6* by VISTA. Red marks conserved non-coding region, light-blue is UTR, and dark-blue indicates exons. Heights of peaks indicate percentage of identify. The regions where the potential Isl1/Pou4f2 binding sites were identified are underlined. B: EMSA with wild-type and mutant probes derived from *Ebf3*. The sequences of wild-type and mutant probes are shown on the top. Lines on top and underneath the wild-type sequences indicate the ATTA cores and Pou4f2 binding motif respectively. The bottom is the EMSA results. Both Isl1 and Pou4f2 could independently bind to the wild-type probe, but not the mutant probe. The Isl1/Pou4f2 complex can bind the wild-type probe, but not the mutant, as indicated by the slow-migrating complex. C: The same experiment as that of B, except that the wild-type and mutant probes were derived from *Irx6*. Both proteins as well as the complex could bind to the wild-type probe, but not the mutant one. D: ChIP analysis of Isl1 and Pou4f2 binding in vivo. Genomic DNA from ChIP by anti-Pou4f2 (P4f2) and anti-Isl1 were amplified by PCR with primers flanking the Isl1/Pou4f2-binding regions of *Ebf3* and *Isl1* and a control region of β-actin. Non-specific IgG was used as control in both ChIP experiments.

Many genes expressed in the developing RGCs are co-regulated by Isl1 and Pou4f2. It is conceivable that many of them are direct targets of these two factors, but the mechanism by which Isl1 and Pou4f2 co-regulate their target genes is unknown, largely due to the fact that few bona fide target genes co-regulated by Isl1 and Pou4f2 have been identified. To identify candidate target genes for Isl1 and Pou4f2, we took a bioinformatics approach by examining binding sites for these two transcription factor in the downstream genes of Isl1 and Pou4f2, identified by microarray [10], [11] and RNA-seq (unpublished). Among the many candidate sites, we focused on two sites found in the upstream regions of *Ebf3* and *Irx6* respectively (**Fig. 7A**), since these two sites have multiple TAAT motifs as observed in SBRN3 and CBRN3 sequences and are highly conserved in vertebrates. We then performed EMSA with radio-labeled double-strand oligonucleotide probes derived from these two motifs with purified Isl1 and or Pou4f2D5. We used Pou4f2D5, instead of full-length Pou4f2, because it is more stable and easier to express and purify. Isl1 could bind both the *Ebf3* and *Irx6* probes as indicated by the retarded band in EMSA (**Fig. 7B,C**), and Pou4f2D5 could bind to both probes as well (**Fig. 7B,C**). However, when the core of the TAAT motifs were changed to other sequences, both Isl1 and Pou4f2D5 failed to bind the mutant probes (**Fig. 7B,C**). As observed with SBRN3 and CBRN3, when both Isl1 and Pou4f2 were present, slower migrating complexes were observed with both *Ebf3* and *Irx6* probes, but no complex formed with the either mutant probes (**Fig. 7B,C**). These results indicate that the Isl1/Pou4f2 complex binds to both the *Ebf3* and *Irx6* motifs.

Next we examined whether Isl1 and Pou4f2 could bind to the *Ebf3* and *Irx6* motifs in vivo in the developing RGCs by ChIP with E14.5 mouse retinal tissues. We chose the stage of E14.5 since this is the time when RGC formation is at the peak, Isl1 and Pou4f2 have the highest expression levels, and most other retinal cell types have not formed. Chromatins were isolated from the retinal tissues, fragmented, and incubated with anti-Isl1 or anti-Pou4f2. Non-specific IgG from the same species (goat) was used as control. DNA was then purified from the chromatins precipitated by the antibodies and amplified by PCR, using primers flanking the identified motifs in the upstream regions of *Ebf3* and *Irx6*. A pair of primers from a non-relevant gene (*β-actin*) was used as control (**Fig. 7D**). Little amplification was observed with DNA samples precipitated by non-specific IgG for the *Ebf3* and *Irx6* fragments, but significant amplifications were observed for both the *Ebf3* and *Irx6* fragments with DNA samples precipitated by both anti-Pou4f2 and anti-Isl1. For the control gene *β-actin*, little amplification could be seen in DNA precipitated by IgG, anti-Pou4f2, or anti-Isl1. These results suggest that Pou4f2 and Isl1 bind to the same DNA regions upstream of *Ebf3* and *Irx6*, and that *Ebf3* and *Irx6* are bona fide target genes of Pou4f2 and Isl1.

### Isl1 and Pou4f2 contribute quantitatively to regulate gene expression in RGCs

**Figure 8:**
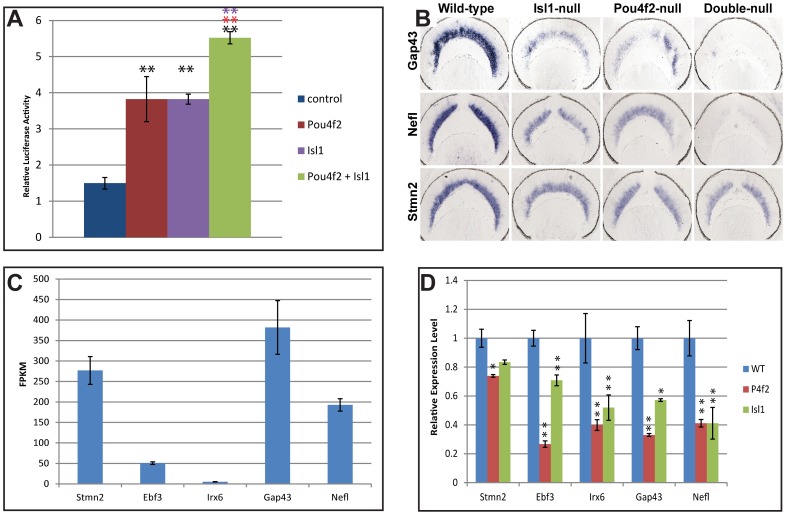
Isl1 and Pou4f2 contribute quantitatively to the full levels of expression of downstream genes. A. Luciferase assays indicating that whereas Pou4f2 and Isl1 can each activate transcription from the reporter alone, together they promote transcription to a higher level. N = 3 for all transfections. Significance of differences was evaluated by student t test. Double black asterisks indicate p<0.01 when compared with control, double red asterisks indicate p<0.01 when compared with Pou4f2, double purple asterisks indicates p<0.01 when compared with Isl1. B. In situ hybridization of three downstream genes, *Gap43*, *Nefl*, and *Stmn2*, of Isl1 and Pou4f2. Compared to the wild-type retina, although all three genes have significantly reduced levels of expression in the RGCs of both *Isl1*- and *Pou4f2*-null retinas, substantial levels of expression remain. In the *Isl1*/*Pou4f2* double null retinas, *Gap43* and *Nefl* are not detectable, but Stmn2 is still expressed. C. The expression levels of downstream genes of Isl1 and Pou4f2 vary greatly. Shown are the relative expression levels of five downstream genes as revealed by RNA-seq. Y axis is Fragments Per Kilobase of exon per Million fragments mapped (FPKM) based on the number of sequence reads and lengths of the transcripts of individual genes. D. Changes of expression levels of five downstream genes of Isl1 and Pou4f2 in the *Isl1*- and *Pou4f2*–null retinas as revealed by RNA-seq. WT: wild-type; P4f2: Pou4f2. The expression levels (Y axis) are calculated based on the FPKM of three replicates and the average values for each gene in the wild-type is set to1. N = 3 for all samples. Significance of differences was evaluated by student's t test. Single asterisk indicates p<0.05, double asterisks indicate p<0.01.

Next we sought to understand the significance of the Isl1/Pou4f2 interaction in regulating downstream genes during RGC formation. We first performed luciferase assay using a reporter construct containing the CBRN3 motif [38]. As reported previously, Pou4f2 increased expression from this reporter construct by around 2.5 fold when a Pou4f2-expressing construct and the reporter construct were co-transfected into HEK 293 cells (**Fig. 8A**) [10], [36], [41]. When an Isl1-expressing plasmid was co-transfected with the reporter construct, an approximately ∼2.5 fold increase of expression was also observed. This was consistent with our finding that Isl1 alone could bind to the CBRN3 motif. When both constructs were co-transfected with the reporter construct, we detected a ∼3.5 fold increase from the control. Therefore, the Isl1/Pou4f2 complex only led to a modest increase from when only Pou4f2 or Isl1 was transfected, suggesting that the collaboration of Pou4f2 and Isl1 was not synergistic. Nevertheless, this further increase in the transcription by the presence of both transcription factors suggests that both are needed to achieve the maximal levels of expression of target genes.

To further examine whether this is the case in the developing RGCs, we analyzed the expression levels of several downstream genes co-regulated by Isl1 and Pou4f2 during RGC development. In situ hybridization analysis of some of these genes indicated that in the absence of either Isl1 or Pou4f2, although their expression is markedly down regulated, significant levels of expression still remained [10], [11], [12]. We confirmed this by examining the expression of three of them, *Gap43*, *Nefl*, and *Stmn2*, by in situ hybridization in *Isl1*-null, *Pou4f2*-null, and *Isl1*/*Pou4f2* double null retinas (**Fig. 8B**). Consistent with previous reports, all three genes were significantly down-regulated, but still had detectable levels of expression in both *Isl1*- and *Pou4f2*-null retinas with the normal spatial expression patterns. However, in Isl1/Pou4f2 double null retinas, there was essentially no expression for *Gap43* and *Nefl*, but a significant level remained for *Stmn2*. These results demonstrate that Isl1 and Pou4f2 contribute quantitatively to the expression of their downstream genes, but their contributions vary in different genes. The substantial remaining levels of expression for these genes in the single knockouts further support the idea that Isl1 and Pou4f2 do not function synergistically. As to *Stmn2*, additional transcription factors must be involved in regulating its expression in the RGCs.

To further quantitatively assess how Isl1 and Pou4f2 contribute to the full-level of expression of the downstream genes they co-regulate, we analyzed how the levels of expression of some these genes changed due to deletion of *Pou4f2* or *Isl1* by examining the RNA-seq data we recently obtained (the full-data set will be published elsewhere). RNA-seq analyzes global expression by sequencing cDNA libraries with next-generation ultra-high-throughput sequencing and mapping the sequence reads to the exons of individual genes. The number of reads mapped to each gene reflects the relative level of expression of the gene in the cells/tissues of interest. The commonly used measure of relative gene expression levels is Fragments Per Kilobase of exon per Million fragments mapped (FPKM) [42]. Our RNA-seq was performed with RNA samples from wild-type, *Pou4f2*-null, and *Isl1*-null retinas at E14.5. The genes we analyzed included *Stmn2*, *Ebf3*, *Irx6*, *Gap43* and *Nefl*, which have all been shown to be downstream of both Isl1 and Pou4f2 by in situ hybridization [11], [12]. Although all expressed in the RGCs at E14.5, they demonstrated a wide-range of expression levels, as their FPKM values ranged from around ∼5 to around ∼400 (**Fig. 8C**). Consistent with previous reports, RNA-seq confirmed that all these genes showed downregulation in both *Pou4f2*-null and *Isl1*-null retinas (**Fig. 8D**). Nevertheless, in agreement with the in situ hybridization data, each gene still maintained considerable expression levels in the mutants, ranging from ∼20% to ∼80% of those in the wild type retina. For each gene, the sum of the remaining levels of expression in the *Pou4f2*-null and *Isl1*-null retinas was either close to or higher than the expression level in the wild-type retinas, indicating that the collaborations of Isl1 and Pou4f2 in regulating these genes were clearly not synergistic, and likely additive, in agreement with our results from the luciferase assays and in situ hybridization.

These results suggest Isl1 and Pou4f2 each alone can substantiate significant levels of expression of downstream genes. Nevertheless, both are required for the normal expression levels, and thereby the normal differentiation of RGCs. However, although Isl1 and Pou4f2 form a complex, they do not collaborate synergistically, underscoring the significance of fine-tuning in regulation of gene expression during development of RGCs.

### Interaction between Lim-Homeodomain and POU domain transcription factors is a common phenomenon

**Figure 9:**
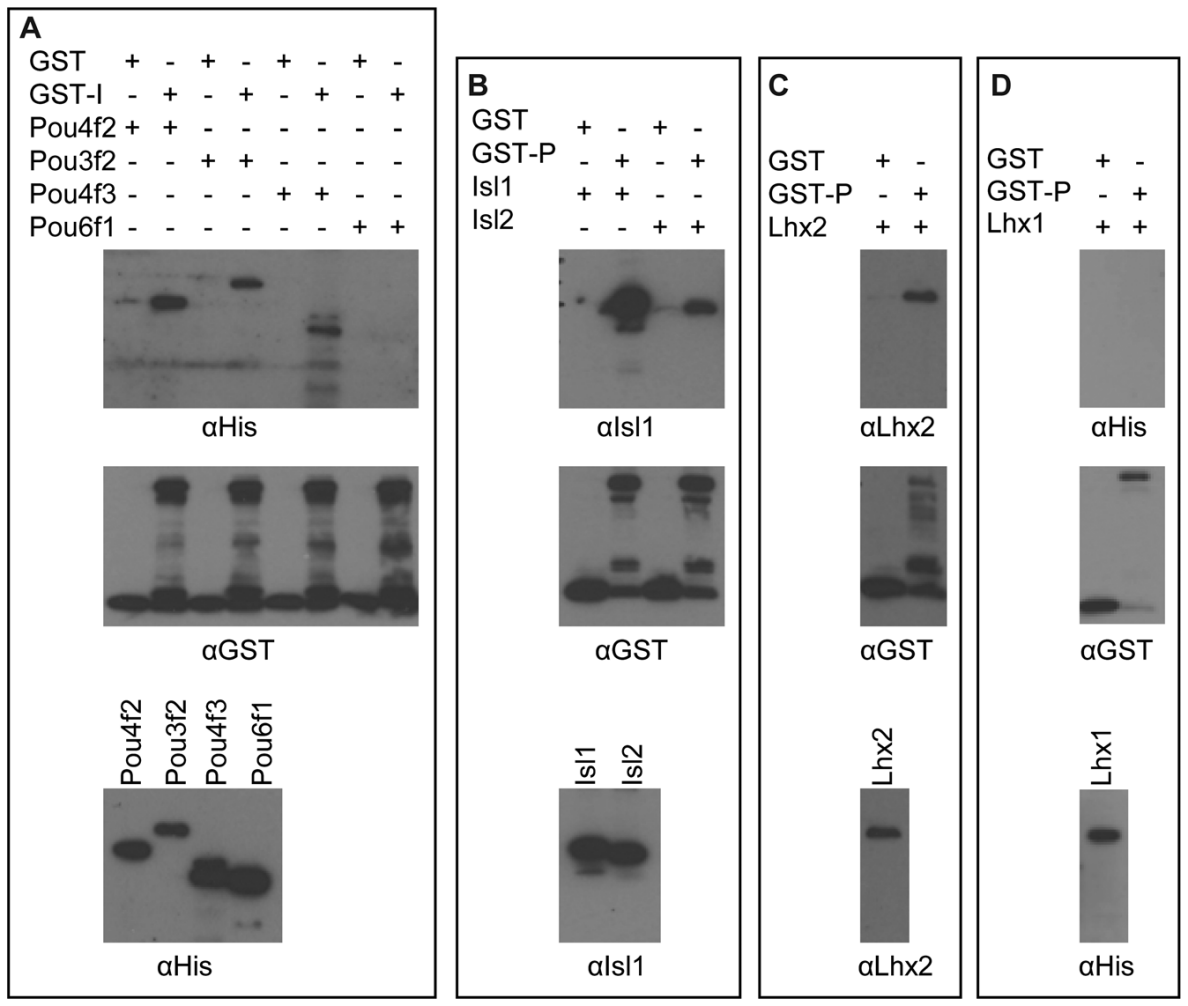
GST pull-down assays show other Lim-Homeodomain and POU-domain proteins interact with Pou4f2 and Isl1 respectively. A: GST-Isl1 (GST-I), but not GST alone, can interact with Pou3f2 and Pou4f3, but not Pou6f1. Pou4f2 was used as positive control. Top: Detection of pulled down proteins by anti-His. Middle: Input GST or GST-Isl1 by anti-GST. Bottom: Input POU-Homeodmain proteins detected by anti-His. B,C,D: GST-Pou4f2 (GST-P), but not GST alone, can interact with Isl2, Lhx2, but not Lhx1. In each panel, the top is detection of pulled down protein by the indicated antibodies, the middle is input GST or GST-Pou4f2 as detected by anti-GST, and the bottom is detection of the input Lim-Homeodomain proteins by the indicated antibodies (anti-Isl1 for Isl1 and Isl2, anti-Lhx2 for Lhx2, and anti-His for Lhx1).

Isl1 and Pou4f2 belong to the Lim-Homeodomain and POU domain transcription factor families respectively. Both families have multiple members and are classified into several subclasses based on sequence similarity [16], [17]. Some members from these two families have been reported to interact physically [19], [20], [38], [43]. These reports and our current finding that Isl1 and Pou4f2 can interact with each other and form a complex indicate that the interaction between the two classes of proteins might be a common phenomenon. To investigate this possibility, we used GST pull-down assay to examine whether Isl1 interacted with other POU domain factors, or vice versa, i.e. whether Pou4f2 interacted with other Lim-Homeodomain factors (**Fig. 9**).

For this purpose, we expressed His-tagged version of Pou3f2 (also known as Brn2), Pou4f3, and Pou6f1 (also known as Brn5) in *E. Coli*. All three proteins were successfully expressed and could be detected by anti-His (bottom panel of **Fig. 9A**). Pou3f2 and Pou6f1 belong to Classes III and VI respectively, whereas Pou4f3 belongs to the same Class IV as Pou4f2. We then examined whether any of them could be pulled down by GST-Isl1. As shown in **Fig. 9A**, whereas the GST control could not pull down any of these proteins, GST-Isl1 pulled down Pou3f2 and Pou4f3, but not Pou6f1, as detected by anti-His. These results indicated that Isl1 could form complexes with Pou3f2 and Pou4f3, but not Pou6f1. It was not surprising that Pou4f3 interacted with Isl1, since Pou4f3 and Pou4f2 belongs to the same class and share high a.a identity in their POU-Homeodmains. The interaction of Isl1 with Pou3f2, but not Pou6f1, indicates that Isl1 forms complexes with selected, but not all, classes of POU domain transcription factors.

We also did the reverse experiment, using GST-Pou4f2 as bait to evaluate whether Pou4f2 could interact with other Lim-Homeodomain proteins. The Lim-Homeodomain transcription factors we assessed included Isl2, Lhx2 and Lhx1. These proteins were successfully expressed and could be detected by anti-Isl1/2, anti-Lhx2, and anti-His antibodies respectively (bottom panels of **Fig. 9B,C,D**). Isl2 is closely related to Isl1, and as expected, was efficiently pulled down by GST-Pou4f2 (**Fig. 9B**). Lhx2 was also efficiently pulled down by GST-Pou4f2, but not by GST alone, as detected by anti-Lhx2 (**Fig. 9C**). On the other hand, although Lhx1 (His-tagged) could be easily detected by anti-His, no signal was observed on the Western blot for the elutant from either GST or GST-Pou4f2 (**Fig. 9D**), indicating that Pou4f2 did not interact with Lhx1. These results suggest that Pou4f2 can interact with selected members of the Lim-Homeodomain protein family as well.

These results suggest that physical interactions between members of the Lim-Homeodomain and POU domain families are prevalent, but not universal.

## Discussion

### Interaction of Pou4f2 and Isl1

Our finding that Pou4f2 and Isl1 physically interact provides a mechanism by which the two transcription factors regulate the downstream genes. Isl1 has been reported to interact with many proteins, and in most cases, the domains involved in protein-protein interaction are the two Lim domains [37], [40]. However, the Lim domains are not involved in the interaction with Pou4f2, since truncated Isl1 lacking the Lim domains are still capable of interacting with Pou4f2. Instead, the homeodomain and the undefined C terminal region are both required for the interaction. There has not been much study regarding the domains of Pou4f2 involved in protein-protein interaction, although the POU-Homeodmains of other POU factors have been implicated in protein-protein interactions [44]. In this study, we found that the POU-Homeodmain and small region adjacent to the POU-Homeodmain on the N terminal side are essential and sufficient for its interaction with Isl1. However, currently we don't know the details of the interaction of these two proteins. Since homeodomains and POU domains are widely involved in both homodimerization and heterodimerization [45], [46], [47], [48], [49] and the regions required for Isl1/Pou4f2 interaction these contain these domains, it is conceivable that they play key roles in their interaction.

### DNA binding by the Isl1/Pou4f2 complex

Both Isl1 and Pou4f2 can bind to DNA individually, yet the two proteins are not always co-expressed, suggesting that the interaction of Isl1 and Pou4f2 is not obligate. Indeed, whereas Pou4f2 is only involved in RGC differentiation, Isl1 ialso plays roles in other retinal cell types such as bipolar cells and amacrine cells [11], [50]. Further, even in the developing RGCs, other than co-regulating a subset of their downstream genes, each transcription factor regulates genes not controlled by the other [11]. Moreover, Isl1 and Pou4f2 seem to be differentially involved in the RGC subtypes [51]. These findings suggest that Isl1, Pou4f2 and the Isl1/Pou4f2 complex may have different DNA-binding properties. Interestingly, the consensus DNA motif bound by Pou4f2 contains the TAAT core sequence that is recognized by Isl1. Consistent with this finding, DNA motifs recognized by Pou4f2 are also bound by Isl1, as well as the Isl1/Pou4f2 complex. Since the DNA motifs studied here contain overlapping motifs recognized by both Pou4f2 and Isl1, an important question that remains is whether each protein in the complex binds to the DNA motifs the same way as when it interacts with the DNA motifs alone. Again this is unlikely since the DNA motif recognized by Isl1 is contained within the motif bound by Pou4f2. Another question that arises is whether all DNA motifs recognized by one factor can be bound by the other one and the complex. This is not likely since many genes are regulated by one factor alone, but not the other. However, to answer this question more definitively, we need to have comprehensive sets of binding sites for both factors in vivo. ChIP-seq analysis in the future will be able to provide this information. Since the DNA-binding domains are involved in the interaction of the two proteins, it is possible that the formation of the Isl1/Pou4f2 complex changes the conformation of the DNA binding domains of each protein, thereby altering their DNA-binding properties in the complex. The complex may also provide new interface(s) which allow for interaction with different co-factors. Both questions will only be addressed by further structural studies.

### Regulation of gene expression in RGCs by the Isl1/Pou4f2 complex

Quantitative analysis of gene expression in *Pou4f2* and *Isl1* knockout retinas indicates that in the absence of one factor, the other can still maintain substantial levels of expression for many downstream target genes. Two important conclusions can be drawn from this observation. First, in the absence of one factor, the other factor may still bind to the DNA motif in the regulatory region of the target gene. This is consistent with our EMSA analysis which shows Isl1 or Pou4f2 alone can bind to the same DNA motif. Second, although the collaboration of Isl1 and Pou4f2 do not seem to be synergistic for the expression of many target genes, both factors are required for their optimal levels of expression, which are crucial for the normal differentiation. This non-synergistic collaboration of Isl1 and Pou4f2 RGCs is very similar to that of Isl1 and Pou4f1, another class IV POU domain transcription factor in the developing dorsal root ganglia and trigeminal ganglia [27]. Isl1 and Pou4f1 may regulate downstream genes in a similar fashion to that of Isl1 and Pou4f2. In other words, Isl1 may be able to form a complex with Pou4f1, just as it does with Pou4f2 and Pou4f3, since all three proteins share a very high a.a. identity in the POU-Homeodmain, although we were unable to examine this due to technical reasons. Since Isl1 and Pou4f2 form a complex, how Isl1 and Pou4f2 regulate downstream genes individually is not clear. As mentioned above, this may be achieved through distinct classes of cis DNA motifs. These motifs may only be recognized by one factor, but not the other or the complex; therefore only one factor is involved in the regulation of the downstream genes. Again, identifying all the binding sites of Isl1 and Pou4f2 will help to answer this question in the future. Although inputs from Isl1 and Pou4f2 contribute quantitatively to the normal levels of target gene expression, they don't seem to regulate the aptitude of expression, as the relative levels of their target genes vary tremendously; other regulatory inputs must be at play to control the expression aptitudes of the many target genes.

### Comparison to the MEC-3/UNC-86 interaction

In *C. elegans*, the interaction of the POU-Homeodomain protein UNC-86 and Lim-Homeodomain protein MEC-3 is essential for the specification of touch receptor neurons [52]. This interaction has been well characterized both genetically and biochemically. Our study revealed that the Isl1/Pou4f2 interaction is analogous to that of MEC-3/UNC-86, but significant differences also exist between the two systems. In both cases, the interaction can take place in the absence of DNA, but both protein complexes regulate target genes by binding to specific DNA motifs [18], [21], [22]. However, although Isl1 alone can efficiently bind the DNA motif recognized by Pou4f2, MEC-3 either does not bind DNA by itself or binds DNA very poorly [18], [21], [22]. Formation of the MEC-3/UNC-86 complex increases the specificity and stability of DNA-binding [22]. This may also be true for the Pou4f2/Isl1 complex, but more detailed studies are needed to confirm this. In addition, unlike the Pou4f2/Isl1 complex, which demonstrates non-synergism in regulating target genes, UNC-86 and MEC-3 activate target genes synergistically [18]. The differences may be due to the fact that although UNC-86 and Pou4f2 belong to the same classes of POU-Homeodomain proteins [16], MEC-3 and Isl1 are distantly related [17]. Nevertheless, the interaction interfaces in UNC-86 and MEC-3 has been identified to be located in their DNA-binding domains [19], [20], [22], which is consistent with our domain-mapping results for the interaction between Pou4f2 and Isl1. Thus, despite of the differences, the two interactions may take place through a shared molecular mechanism to regulate expression of downstream genes.

### Interaction between members of the Lim-Homeodomain and POU domain families

Our finding that Isl1 and Pou4f2 interact with other members of POU domain and Lim-Homeodomain transcription factors respectively indicate that the interaction of these two families of transcription factors is not just confined to Isl1 and Pou4f2 but common between members of these two families, and that interaction of these two types of transcription factors is conserved during evolution. This is supported by the interaction of MEC-3 and UNC-86 in *C. elegans*. Although UNC-86 and Pou4f2 belong to the same subclass of POU domain factors, MEC-3 and Isl1 are not as closely related [16], [17]. Each factor therefore seems to be able to interact with members of several different classes of the other family. Studies on other members of these two families also support the idea that interactions between members from these two families are prevalent. However, it is clear that not all members between these two families interact, indicating that certain degree of selectivity and specificity exists. Currently, it is not known what determines this specificity. Since there are multiples subclasses and members of both families of transcription factors and they are expressed and function in diverse developmental and other biological processes [16], [17], it is conceivable that additional interactions between the two families exist. The interactions between members of these two families of transcription factors generate additional diversity of regulatory inputs in regulating gene expression.
